# Loneliness, Social Support, & Perceived Economic Hardship in Self-Management of Older Liver Transplant Recipients

**DOI:** 10.1093/geroni/igaf122.4157

**Published:** 2025-12-31

**Authors:** JiYeon Choi, JaeWon Hyun, Bomgyeol Kim, Hun Kang, Jin-kyung Lee

**Affiliations:** Yonsei University, Seoul, Republic of Korea; Yonsei University, Seoul, Republic of Korea; Yonsei University College of Nursing, Seoul, Republic of Korea; Yonsei University, Seoul, Republic of Korea; Yonsei University College of Nursing, Seoul, Republic of Korea

## Abstract

Liver transplantation is a highly selective and resource-intensive procedure that challenges both individuals and health systems. With more older adults receiving liver transplantation to treat end-stage liver disease, it is important to recognize that outcomes are shaped not only by aging-related physical and cognitive challenges but also by psychosocial factors, including loneliness, social support, and perceived economic hardship. We examined relationships among loneliness, perceived social support, perceived economic hardship, and self-management in older liver transplant recipients (LTRs) (age≥60), focusing on the mediating role of perceived social support and the moderating role of perceived economic hardship. A cross-sectional analysis was conducted using data from 131 LTRs (Age 65.24±3.91 years, 55.8% men, 27.78±17.00 months post-transplant) recruited from a tertiary academic medical center in South Korea. Measures included Loneliness scale (3-item), Multidimensional Scale of Perceived Social Support (12-item), Modified Transplant Self-management Scale (16-item), and economic hardship (one-item). Perceived economic hardship was classified into three categories: difficult, moderate, and none. Moderated mediation analysis was conducted using Hayes’ PROCESS macro (Model 15), controlling for gender, income, education, marital status, living arrangement, return to work status, cognitive status, and frailty. Loneliness was associated with lower perceived social support (β=–0.27, p=.002), and higher social support was associated with better self-management (β = 5.91, p<.001). The indirect effect of loneliness on self-management via social support was significant across perceived economic hardship levels (index=0.503, 95% CI 0.03-1.04). Findings suggest that financial constraints may diminish the protective role of social support. Interventions to enhance self-management should address psychosocial barriers in aging LTRs.

